# Editorial for “Characteristics of right pulmonary vein with epicardial connection needing additional carina ablation for isolation”

**DOI:** 10.1002/joa3.12985

**Published:** 2024-01-02

**Authors:** Kentaro Yoshida

**Affiliations:** ^1^ Department of Cardiology Ibaraki Prefectural Central Hospital Kasama Japan; ^2^ Department of Cardiology, Institute of Medicine University of Tsukuba Tsukuba Japan

**Keywords:** atrial fibrillation, epicardial connection, intercaval bundle, pulmonary vein isolation

More than a quarter century has passed since the introduction of catheter ablation of atrial fibrillation (AF). Although a number of strategies targeting the arrhythmogenic substrate beyond pulmonary veins (PVs), such as ablation of continuous fractionated atrial electrograms and linear ablations, have been advocated, the only established strategy currently supported by randomized clinical trials is isolation of the PVs. Nevertheless, reconnections of PVs are still common during and after the procedures, resulting in the need for a significant number of redo procedures. This is a critical issue from the economic point of view for society and both mental and physical aspects for the patients.

One of the reasons for this high prevalence of PV reconnections was recently considered to be the three‐dimensional complex structures of the left atrium and PVs, mainly represented by epicardial muscular bundles, that is, the Marshall bundle, septopulmonary bundle, and intercaval bundle. Isolation of the right‐sided PVs seems to be more commonly affected by epicardial connections compared to left‐sided PV isolation, and the intercaval bundle posteriorly connecting both the left and right atrium is the most common fiber/bundle making isolation of right‐sided PVs difficult.^1^, ^2^ Although it is difficult to directly prove the involvement of this bundle in human PVs, there may be strong evidence that ablation in the posteroseptal wall of the right atrium occasionally can isolate the right‐sided veins.^3^, ^4^ Because this bundle is recognized in most (~90%) of the population on anatomical studies, the relative location of this fiber in relation to the PVs, its insertion sites in the PVs or atrium, the depth and orientation of this fiber, and conduction direction and velocity, may determine whether it actually precludes right‐sided PV isolation. For example, if this fiber runs endocardially in the epicardial layer, its insertion is outside the antral ablation line and its conduction is weak and slow, making it easily ablated by routine antral ablation encircling the PVs. In this regard, the design of an anterior line during right‐sided antral ablation that is mainly a certain distance from the PV ostium is an important factor.^5^ Therefore, it is not surprising that anatomical characteristics and features of the left atrium and PVs could be predictors for preclusion of first‐pass isolation because of epicardial connections.^6^

In this issue of the *Journal of Arrhythmia*, Nehashi et al. focused on the anatomical features of the right‐sided PVs using computed tomography scans and found that the right‐sided PV bifurcation angle and the area occupation ratio of the right PV carina region were associated with the requirements of carinal ablation for PV isolation.^1^ When we see a wider PV bifurcation angle and wider area of the carinal region, first‐pass isolation may be more difficult and carinal ablation may be required to isolate the PVs. This was a retrospective, cross sectional study, and therefore, changes in the anatomy of the right and left atrium and PVs because of structural remodeling in AF and in their relative structural relation were not included in the analyses. The underlying mechanisms for the relation between such an anatomical characteristics and the contribution of epicardial connections is also unclear. Although this study did not focus on the rhythm outcomes after ablation, our therapeutic goal is currently not to isolate PVs during the procedures but to decrease redo procedures for which epicardial structures may be the most important contributor, and I hope that imagining the invisible epicardial structures in our mind by relying on the predictive anatomical and electrophysiological factors will prevent reconnections of PVs and improve clinical outcomes. In addition to the intercaval bundle, the coronary sinus, Marshall bundle, and septopulmonary bundle are also important epicardial structures in linear ablations and PV isolation, but anatomical predictors remain to be fully explored.

The intercaval bundle may be a physiologically important structure with characteristic functional properties in interatrial conduction during sinus rhythm similar to those of the Bachmann bundle and in the development of atrial tachycardia and AF. In my experience, epicardial conductions in the severely remodeled atrium will likely be manifested and pronounced during sinus rhythm propagation, as shown in a case report.^7^ As a landmark first report showing the clinical importance of this bundle, a four case series of patients with biatrial tachycardia involving the intercaval bundle was reported.^4^ We also reported two cases of iatrogenic parasystole in the right atrium after PV isolation that depended on unidirectional conduction through this bundle.^3^ I believe that the unidirectional conduction property may not be coincident but specific for epicardial connections such as in WPW syndrome and may contribute to the high likelihood of biatrial macro‐reentry and parasystole. Hasebe et al. recently investigated the frequency gradient during AF in patients with and without dominant epicardial conduction.^8^ Because drivers are commonly present in the PVs and left atrium, it is well known that a frequency gradient from the left to right is present especially in paroxysmal AF. Conduction through the intercaval bundle may diminish this gradient and hierarchy and might enhance electrical remodeling in AF. Taken together, the intercaval bundle is embryologically, anatomically, functionally, pathophysiologically, and therapeutically an interesting structure for electrophysiologists performing ablation of atrial tachyarrhythmias. I have been interested in these connections between atrial chambers and cardiac veins from 2016 and am thankful and proud that a number of case reports, case series, and articles with detailed evaluation of electrophysiological findings have recently been published from Japan. Although future advancements in technologies such as pulsed‐field ablation may resolve the issues of PV reconnections and failed linear ablation because of these epicardial bundles, further investigation from the electrophysiological points of views should be also important to better understand the electrophysiology in three‐dimensional complex structures of the atrium from both the physiological and pathophysiological aspects.

## CONFLICT OF INTEREST STATEMENT

Authors declare no conflict of interests for this article.

## References

[1] Nehashi T , Kaneshiro T , Nodera M , Yamada S , Takeishi Y . Characteristics of right pulmonary vein with epicardial connection needing additional carina ablation for isolation. J Arrhythm. 2023;39(6):884–893. 38045469 10.1002/joa3.12944 PMC10692864

[2] Yoshida K , Baba M , Shinoda Y , Harunari T , Tsumagari Y , Koda N , et al. Epicardial connection between the right‐sided pulmonary venous carina and the right atrium in patients with atrial fibrillation: a possible mechanism for preclusion of pulmonary vein isolation without carina ablation. Heart Rhythm. 2019;16(5):671–678. 30465905 10.1016/j.hrthm.2018.11.017

[3] Yoshida K , Hasebe H , Hattori M , Hanaki Y , Tsumagari Y , Baba M , et al. Unidirectional conduction characterizing epicardial connections in patients with atrial tachyarrhythmias. J Cardiovasc Electrophysiol. 2023;34(11):2262–2272. 37712297 10.1111/jce.16065

[4] Patel PJ , D'Souza B , Saha P , Chik WW , Riley MP , Garcia FC . Electroanatomic mapping of the intercaval bundle in atrial fibrillation. Circ Arrhythm Electrophysiol. 2014;7(6):1262–1267. 25516583 10.1161/CIRCEP.114.001738

[5] Lin YJ , Tsao HM , Chang SL , Lo LW , Tuan TC , Hu YF , et al. The distance between the vein and lesions predicts the requirement of carina ablation in circumferential pulmonary vein isolation. Europace. 2011;13(3):376–382. 21227954 10.1093/europace/euq500

[6] Hanaki Y , Yoshida K , Baba M , Hasebe H , Takeyasu N , Nogami A , et al. Interatrial distance predicts the necessity of additional carina ablation to isolate the right‐sided pulmonary veins. Heart Rhythm O2. 2020;1(4):259–267. 34113879 10.1016/j.hroo.2020.08.003 PMC8183890

[7] Tsumagari Y , Yoshida K , Baba M , Hasebe H . Epicardial connections as intra‐atrial conduction routes in a patient with advanced atrial remodeling. JACC Case Rep. 2021;3(16):1774–1779. 34825208 10.1016/j.jaccas.2021.08.025 PMC8603051

[8] Hasebe H , Yoshida K , Nogami A , Furuyashiki Y , Ieda M . Impact of interatrial epicardial connections on the dominant frequency of atrial fibrillation. Circ J. 2023;87(7):973–981. 37258220 10.1253/circj.CJ-22-0769

